# Bladed and bladeless conical trocars do not differ in terms of caused fascial defect size in a Porcine Model

**DOI:** 10.1007/s00464-022-09401-9

**Published:** 2022-07-18

**Authors:** Christoph Paasch, Anne Mantke, Richard Hunger, Rene Mantke

**Affiliations:** 1 Clinic for General and Visceral Surgery, University Hospital Brandenburg an der Havel, Brandenburg Medical University, Hochstraße 29, 14770 Brandenburg an der Havel, Germany; 2 Faculty of Health Sciences Brandenburg, Brandenburg Medical School Theodor Fontane, Brandenburg, Germany

**Keywords:** Porcine Model, Trocar site hernia, Bladed trocar, Bladeless trocar, Incisional hernia

## Abstract

**Introduction:**

Trocar insertion during laparoscopy may lead to complications such as bleeding, bowel puncture and fascial defects with subsequent trocar site hernias. It is under discussion whether there is a difference in the extent of the trauma and thus in the size of the fascia defect between blunt and sharp trocars. But the level of evidence is low. Hence, we performed a Porcine Model.

**Methods:**

A total of five euthanized female pigs were operated on. The average weight of the animals was 37.85 (Standard deviation SD 1.68) kg. All pigs were aged 90 ± 5 days. In alternating order five different conical 12-mm trocars (3 × bladeless, 2 × bladed) on each side 4 cm lateral of the mammary ridge were placed. One surgeon performed the insertions after conducting a pneumoperitoneum with 12 mmHg using a Verres’ needle. The trocars were removed after 60 min. Subsequently, photo imaging took place. Using the GSA Image Analyser (v3.9.6) the respective abdominal wall defect size was measured.

**Results:**

The mean fascial defect size was 58.3 (SD 20.2) mm^2^. Bladed and bladeless trocars did not significant differ in terms of caused fascial defect size [bladed, 56.6 (SD 20) mm^2^ vs. bladeless, 59.5 (SD 20.6) mm^2^*, p* = 0.7]. Without significance the insertion of bladeless trocars led to the largest (Kii Fios™ First entry, APPLIEDMEDICAL©, 69.3 mm^2^) and smallest defect size (VersaOne™ (COVIDIEN©, 54.1 mm^2^).

**Conclusion:**

Bladed and bladeless conical 12-mm trocars do not differ in terms of caused fascial defect size in the Porcine Model at hand. The occurrence of a trocar site hernia might be largely independent from trocar design.

In 1901, the German surgeon Georg Kelling from Dresden (1866–1945) performed a laparoscopic surgery on a dog for the first time. He created the pneumoperitoneum with trocars [1]. Since the first conducted laparoscopic organ resection, a salpingectomy, was performed in 1975 by Tarasconi [2], the trend towards frequent laparoscopic surgery has continued. As is well known, these include laparoscopic cholecystectomy and appendectomy, which are amongst the most frequently performed surgical procedures worldwide [3, 4]. Less pain, faster recovery and lower risk of incisional hernias are revealed advantages over open abdominal surgery [5, 6].

As the number of laparoscopic surgical procedures increases each year, so do the complications (especially bleeding, incisional hernias and visceral damage) due to the laparoscopic access with trocars and Verres’ needle. The literature reports a complication rate of 3–16% [7].

The impact of different port designs (bladed vs. bladeless) on the complication rate have been frequently discussed in literature [8–10]. To that, the International Endohernia Society recommended the insertion of radially expanding bladeless trocars to reduce port-site bleeding [11]. Although it is known that trocars cause persistent fascial defects [9], current guidelines do not recommend which trocar design should be preferred to prevent trocar hernias. This circumstance might reflect the low level of evidence in the literature [11].

To investigate the influence of bladed and bladeless trocars on the fascial defect size, we performed the Porcine Model at hand. The abdominal wall of pigs also consists of M. obliquus ext., int., transversus abdominis and rectus abdominis.

## Methods

The study at hand took place in Beichlingen (Association for the Promotion of Innovative Medicine, Altenbeichlinger Str. 157, 99625 Beichlingen, Germany). Permission for an animal experiment was not obtained. According to §7 of the German Animal Welfare Act, the killing of an animal is not considered an animal experiment if the killing is done exclusively to use the animal’s organs or tissues for scientific purposes [12].

The abdominal wall of pigs consists (like in humans) of the M. obliquus externus, internus, transversus abdominis and rectus abdominis. Therefore, a total number of five masthybrid pigs were used for the examination at hand. The average weight of the animals was 37.85 (SD 1.68) kg. All pigs were female and aged 90 ± 5 days (Table 1).

**Table 1:** Animal biometrics

Animal	Sex	Age (days)	Weight (kg)
I	♀	90 ± 5	41.9
II	♀	90 ± 5	36.2
III	♀	90 ± 5	36.3
IV	♀	90 ± 5	40.2
V	♀	90 ± 5	38.7

**Fig. 1:**
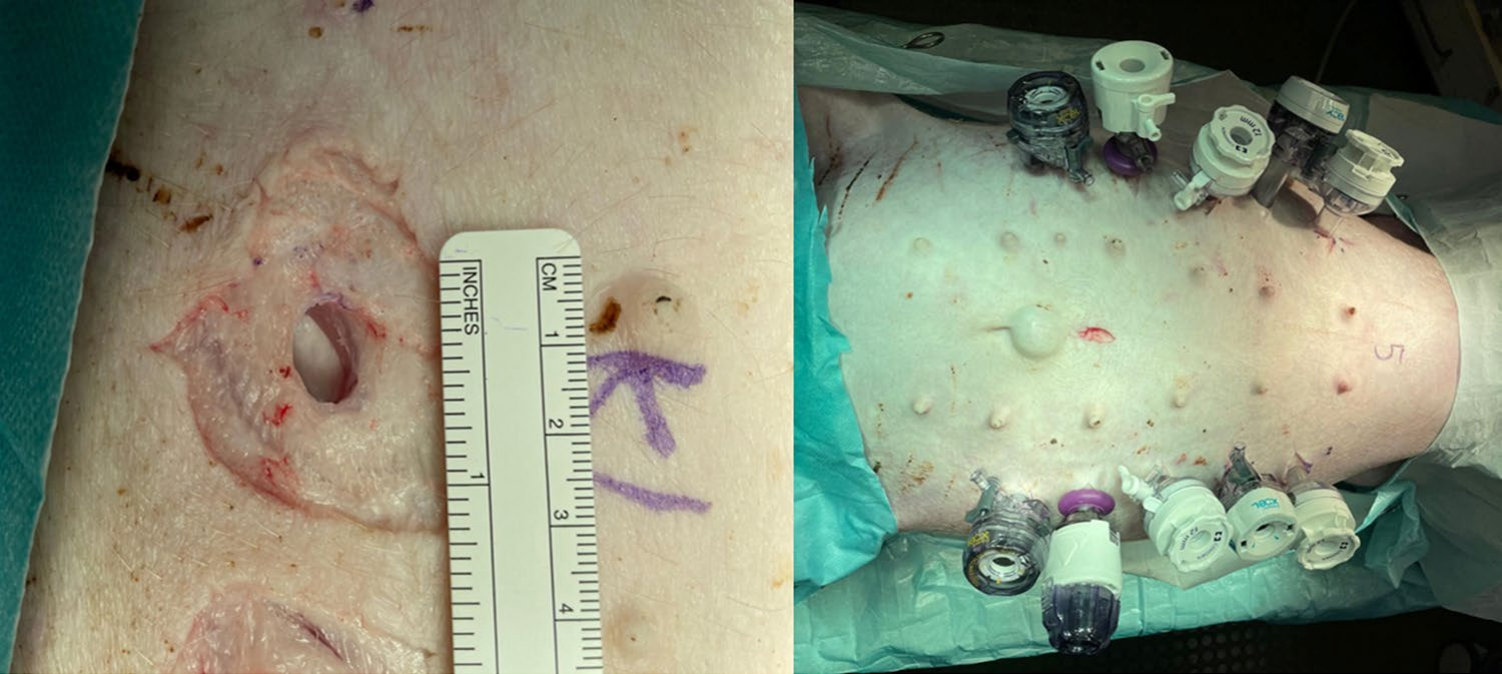
Magnified defect size (left sided) and successfully placed 12-mm trocars (right sided) are depicted

**Fig. 2:**
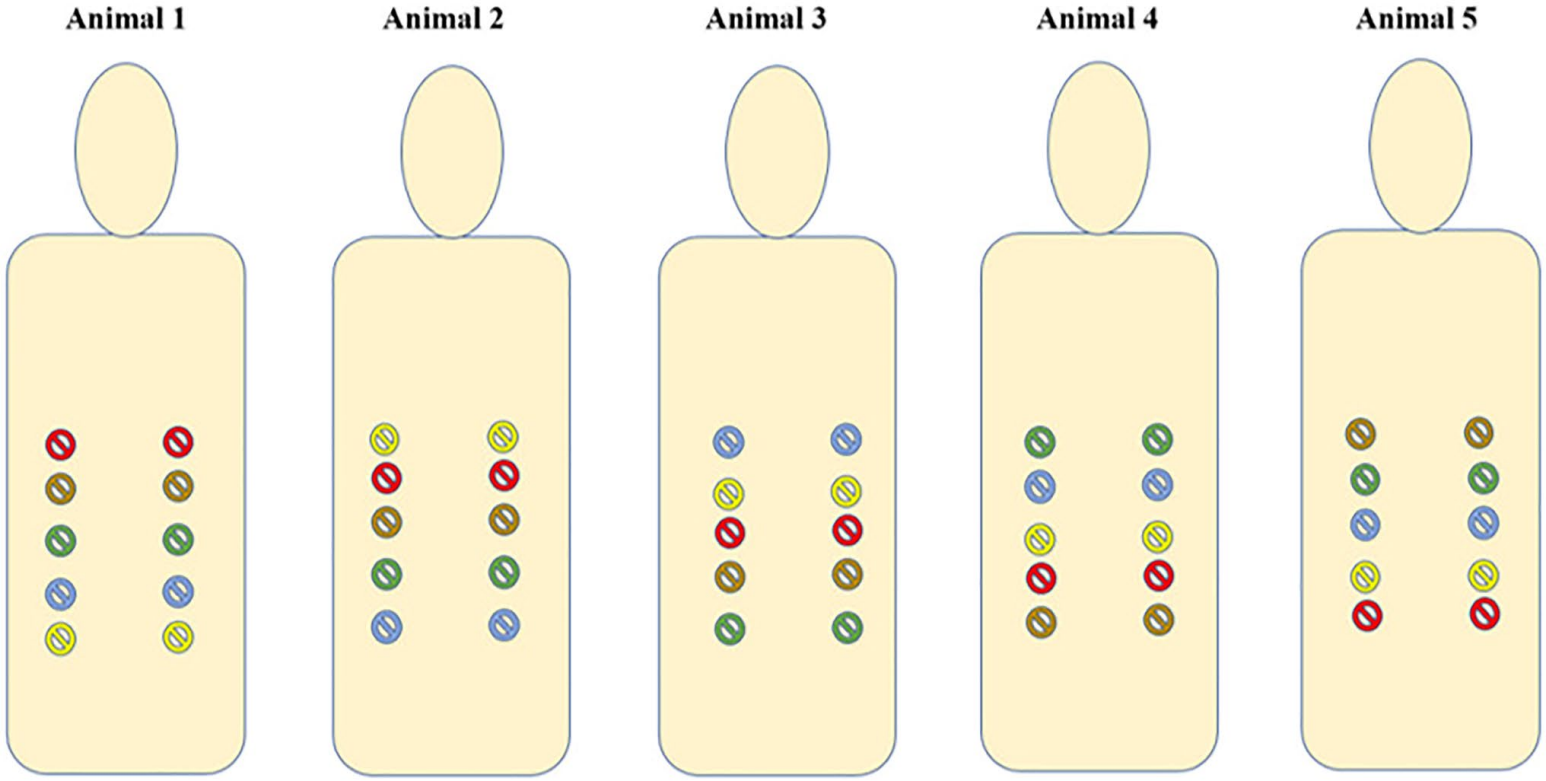
The conical 12-mm trocars were place in alternating order in each animal

The animals were euthanized with the short-acting barbiturate Pentobarbital. Within 10 min a pneumoperitoneum with 12 mmHg for 1 h was conducted after trocar placement (Fig. 1). A total of five different conical 12-mm trocars on each side 4 cm lateral of the mammary ridge were placed. Between two trocars a 5 cm distance was kept. The trocars were place in alternating order in each animal (Fig. 2). The insertion was performed with repetitive right quarter turning. Each individual trocar type was placed 10 times (2/animal). One experienced surgeon (years of work: > 30 years) performed all trocar insertions.

As bladed trocars Bladed VersaOne™ (COVIDIEN©; B12STF, conical tip) and ENDOPATH XCEL™ (ETHICON ENDO-SURGRY©; D12LT, conical tip) were used. The bladeless ports were Bladeless VersaOne™ (COVIDIEN©; NONB12STF, conical tip), ENDOPATH XCEL™ (ETHICON ENDO-SURGRY©; B12LT, conical tip) and Kii Fios™ First entry (APPLIEDMEDICAL©; CFF73, conical tip).

Before the trocar removal, the surrounding skin and fat tissue was removed with a scalpel. The photo imaging was conducted after a ruler was placed next to the wound by one surgeon. The fascial defects (mm^2^) were measured using the GSA Image Analyser v3.9.6 © 2014.

### Statistical analysis

Initially we compared mean defect size between scalpel-trocar-types with a classical *t* test (defect size was normally distributed within and had equal variances across scalpel-trocar-type conditions). To account for repeated measurements (ten measurements per animal) we fitted a linear mixed model using restricted maximum likelihood estimator. The scalpel-trocar-type was entered as fixed and animal as random effect. We hypothesized that there should be no significant main effect of scalpel-trocar-type. In a sensitivity analysis, the different trocar types (bladed vs. bladeless) were additionally included as fixed main effect to model the defect size. A *p* value less than 0.05 was considered as statistical significant.

Due to the exploratory study design and lack of published studies on this topic, no a-priori sample size calculation was performed.

## Results

The mean fascial defect size was 58.6 (SD 20.9) mm^2^.

The insertion of the bladeless trocar and Kii Fios™ first entry (APPLIEDMEDICAL©) led to a defect size of 69.3 (SD 21.7) mm^2^. The placement of the bladeless trocar ENDOPATH XCEL™ (ETHICON ENDO-SURGRY©) caused a defect size of 55.0 (SD 18.3) mm^2^. The insertion of the bladeless VersaOne™ (COVIDIEN©) led to a defect size of 54.1 (SD 20.0) mm^2^.

The placement of the bladed trocar ENDOPATH XCEL™ (ETHICON ENDO-SURGRY©) caused a defect size of 58.1 (SD 22.4) mm^2^. The insertion of the Bladed VersaOne™ (COVIDIEN©) led to a defect size of 56.7 (SD 22.4) mm^2^ (Table 2).

**Table 2:** Descriptive analysis on defect size caused by different trocars

	Defect size (mm^2^)
*Bladed trocars*	
Bladed VersaOne™ (COVIDIEN©; B12STF, conical)	56.7 (22.4)
ENDOPATH XCEL™ (ETHICON ENDO-SURGRY©; D12LT, conical)	55.0 (18.3)
*Bladeless trocars*	
Kii Fios™ First entry (APPLIEDMEDICAL©; CFF73, conical)	69.3 (21.7)
ENDOPATH XCEL™ (ETHICON ENDO-SURGRY©; B12LT, conical)	55.0 (18.3)
Bladeless VersaOne™ (COVIDIEN©; NONB12STF, conical)	54.1 (20.0)
	*p* = 0.5*

*Kruskal–Wallis rank sum test; () Standard deviation

### Box plot analysis

**Fig. 3:**
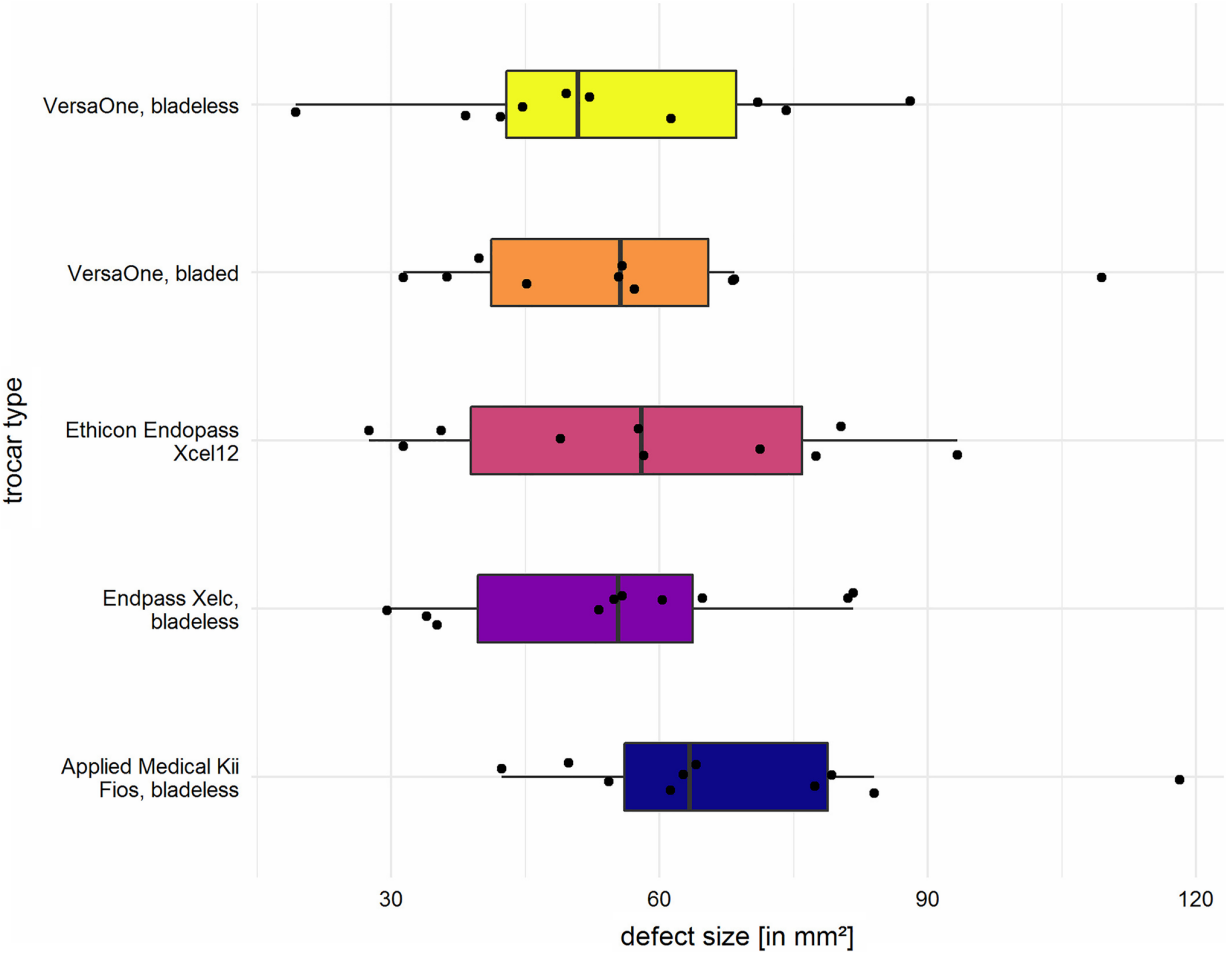
Box plot of defect size stratified by five different conical 12-mm trocars

Smallest mean defect size was observed for the bladeless VersaOne port (COVIDIEN©) with 54.1 mm^2^ (SD 20.0). The insertion of the bladeless trocar Kii Fios™ first entry (APPLIEDMEDICAL©) led to the largest average defect size with 69.3 mm^2^ (SD 21.7) (Table 2 and Fig. 3). The linear mixed model, however, indicated no significant differences between trocar types (*p* = 0.5). Results of the sensitivity analysis yielded virtually identical results.

**Fig. 4:**
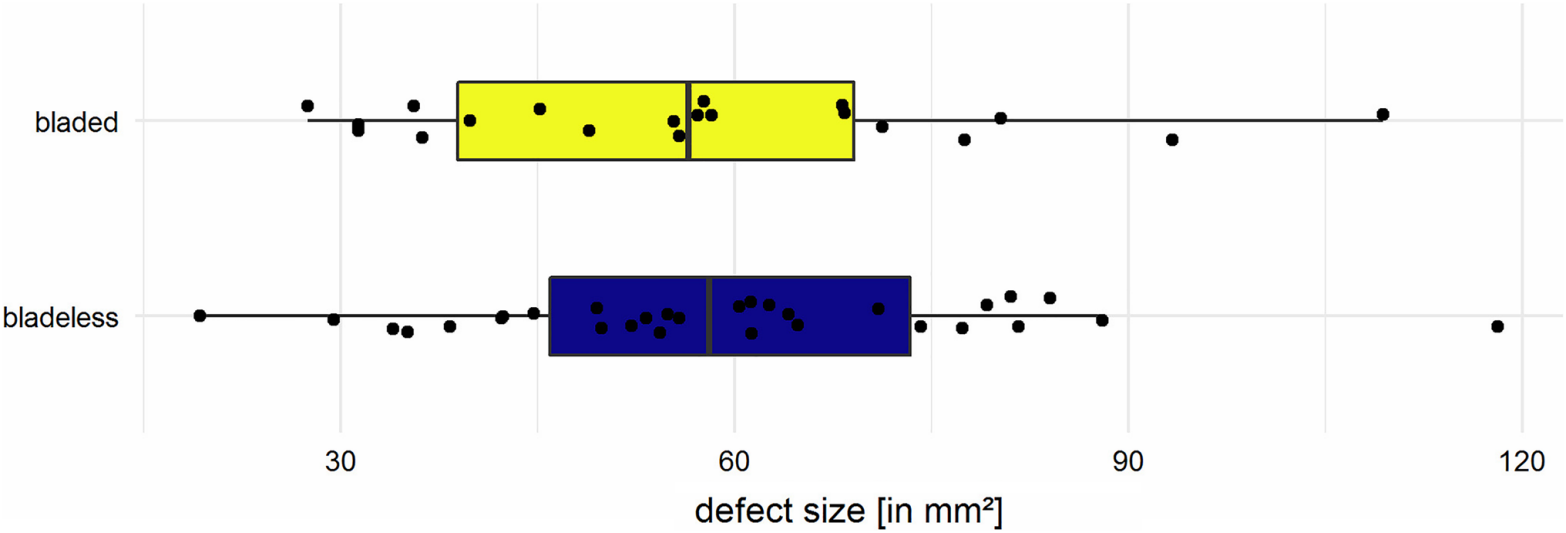
Box plot of defect size caused by bladed and bladeless trocars

Group comparison of defect size between bladed [56.6 (SD 20.0) mm^2^, 20 trocar positions] and bladeless [59.5 (SD 20.6) mm^2^, 30 trocar positions] trocars revealed no significant differences [*t*(48) = 0.34, *p* = 0.7; Table 3 and Fig. 4].

**Table 3:** Wilcoxon rank sum exact test on different trocar types

	Defect size (mm^2^)
Overall (*N* = 50)	58.6 (20.9)
Bladed trocars (*N* = 20)	57.4 (21.8)
Bladeless trocars (*N* = 30)	59.5 (20.6)
	*p* = 0.7

*N* Amount of trocar placement; () Standard deviation

**Fig. 5:**
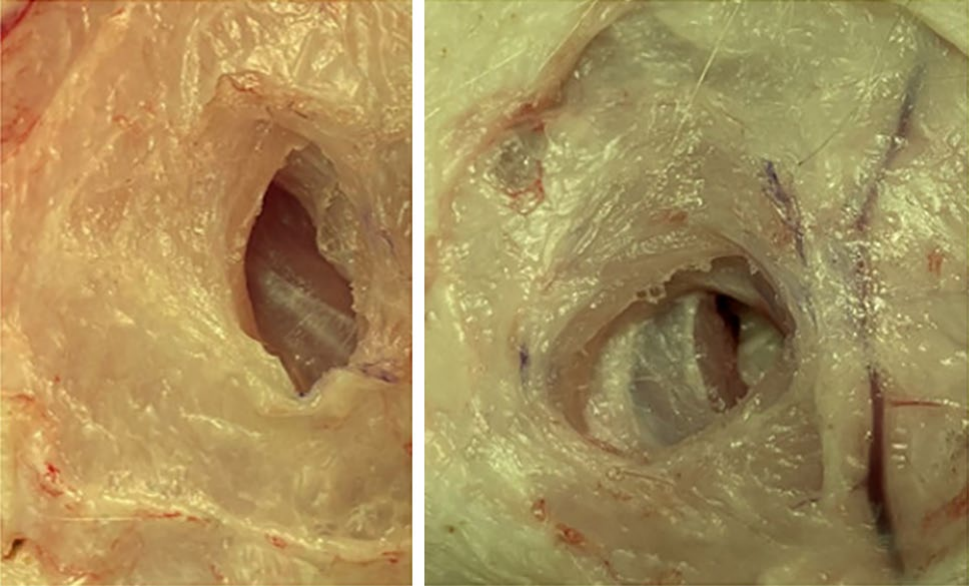
Caused defect size after placement of bladed VersaOne™ (COVIDIEN©). The trocar caused in the same position and animal a 26% difference of the defect sizes (pig V: 93.21 mm^2^, 4 cm left-sided of the mammary ridge; 68.20 mm^2^, 4 cm right-sided of the mammary ridge)

The linear mixed model yielded a statistically non-significant effect of scalpel-type [bladeless vs. bladed, beta = 2.05, 95% CI (− 10.18, 14.28), *p* = 0.738]. The effect of the scalpel-type was amplified in the sensitivity analysis. But it remained non-significant (*p* = 0.19) (see Fig. 5).

## Discussion

The trocar placement during laparoscopic surgery results in fascial defects. These abdominal wall injuries may subsequently cause so called trocar site hernias [13], already described in 1968 [14]. An incidence of 0–5.2% was published by Helstrand et al. after a systematic review of the literature (22 studies, *n* = 31,666 [13]).

It can be postulated that the defect size depends not only on the trocar size but also on the trocar design. Radially expanding bladeless trocars do not have any tissue-cutting features. Hence, it has been assumed that these trocars have the perceived advantage of causing less tissue damage and thus fewer trocar site hernias [10]. To that, Bhoyrul et al. used conventional and radially expanding bladeless trocars in laparoscopic cholecystectomies on 12 pigs. The authors measured a statistical significant narrower defect, when using these bladeless trocars (about 50% narrower, *p* < 0.001) [15]. In addition, Johnson et al. published a retrospective analysis on 747 individuals, who underwent laparoscopic Roux-en-Y gastric bypass surgery. The authors inserted a total of 1494 12-mm bladeless VersaStep™ trocars. No trocar site hernia occurred during a follow-up of 20 (4–43) months [16]. These findings have been confirmed by Gutierrez et al. when conducting a review of literature (Incidence of trocar site hernia: 12-mm bladed (*n* = 710) 1.549% vs. 12-mm bladeless (*n* = 8304) 0.12%, *p* < 0.05) [17]. However, amongst the publications reviewed, the authors found no prospective randomized trials and the compared group were numerously unbalanced.

In contrast, Pilone et al. detected no trocar site hernias in areas, where 12-mm bladed trocars were inserted. The authors used 12-mm ports during bariatric surgery amongst 624 patients [18]. In addition, Erdas et al. did not identify 12-mm bladed trocars as an independent risk factor for trocar site hernias. They conducted a retrospective analysis on 313 individuals, who underwent laparoscopic cholecystectomy [19]. To that, the bladeless trocar Kii Fios™ first entry (APPLIEDMEDICAL©) caused the largest defect size in our study, but without significance.

We did not reveal any differences in terms of caused fascial defect size in our Porcine Model. The 12-mm bladed trocars caused a mean defect size of 57.4 (21.8) mm^2^ and the 12-mm bladeless trocars 59.5 (20.6) mm^2^ (*p* = 0.74). Moreover, the effect of the scalpel-type was amplified in the sensitivity analysis. But remained non-significant (*p* = 0.19). In conclusion, it remains unclear whether trocars with or without blades should be preferred to prevent trocar site hernias. Literature results are contradictive. Furthermore, there is frequently no information on shape of the tip (conical, pyramidal), which makes comparison even more difficult [8, 19]. Most likely, therefore, there is no recommendation on this in the current guidelines [11]. Our results suggest that the fascial defect size might be largely independent from trocar design (bladed vs. bladeless).

As another explanation for different defect sizes might be the angle of trocar and abdominal wall during the insertion. We detected a 26% difference of defect sizes, when the bladed VersaOne™ (COVIDIEN©) trocar was placed in the same animal and in the same position. A different angle during placement is conceivable. On one hand, the defect might be larger, when the angle of trocar and abdominal wall is lower and not right-angled. On the other hand, an acute angle would lead to in parallel located defects of the subsequent layers. Each defect would be covered. But it remains unclear due to lack of evidence.

Other trocar-associated complications are consisting of organ puncture, pain and bleeding [8]. It has been assumed, that bladed trocars may lead to a higher rate of organ puncture [8]. But a higher force for penetration is needed, when using bladeless ports [20]. Although these trocars do not have a cutting surface, viscera may be injured by blunt trauma. To that, a systematic review and meta-analysis (8 randomized trials, *n* = 720) did not elaborate advantages of bladeless over bladed trocars. But the authors revealed a statistical significant lower rate of bleeding after bladeless trocar insertion [10]. On the other hand, a Cochrane analysis from 2015 stated a very low quality of evidence for this assumption. It remains controversial and future trials are mandatory [9].

Beyond the question of whether trocars are used with or without a blade, the shape of the tip, i.e. conical or pyramidal, may play an important role in the development of the fascial defect. To facilitate a sufficient comparison between bladed und bladeless trocar, we decided to insert only conical trocars (Table 2). As already mentioned, information on the shape of the trocar tip is rarely found in literature [8, 19]. But some knowledge on that was revealed by Böhm et al. and Moreno et al. in Porcine Models. The authors came to contradictive results. Böhm et al. stated, that pyramidal bladed and bladeless trocars caused a larger fascial defect than conical bladed and bladeless trocars [21]. On the other hand, Moreno et al. revealed no significant differences on that [20]. Unfortunately, we were not able to compare our results with these Porcine Models, as the authors stated only diameters of the fascial defects and not the square millimetre of the area [20, 21].

Our review of literature yielded to comparable Porcine Models (Shafer et al. eight operated vivid female pigs; Bhoyrul et al. 12 operated vivid female pigs [15, 22]). Different 12-mm conical shaped bladed and bladeless trocars were inserted. In contrast to our findings the authors of both publications stated, that bladed trocars led to a larger fascial defect size with significance. Contrary to our digital evaluation of defect size (GSA Image Analyser v3.9.6 © 2014) no measurement of square millimetre took place. Unfortunately, no detailed information on methods of that has been provided by Shafer et al. [22]. It can be assumed, that the authors measured length × width. Bhoyrul et al. described their defect measurement (length × width) but when analysing the depicted images in this publication, one could argue, that the muscle defect size was evaluated and not the fascial defect. This layer appears to be removed. In their Porcine Model only 24 defects were measured. We analysed 50 defects. In summery these Porcine Models are only to a certain extent comparable. The contradictive results may reflect the need for further studies.

**Fig. 6:**
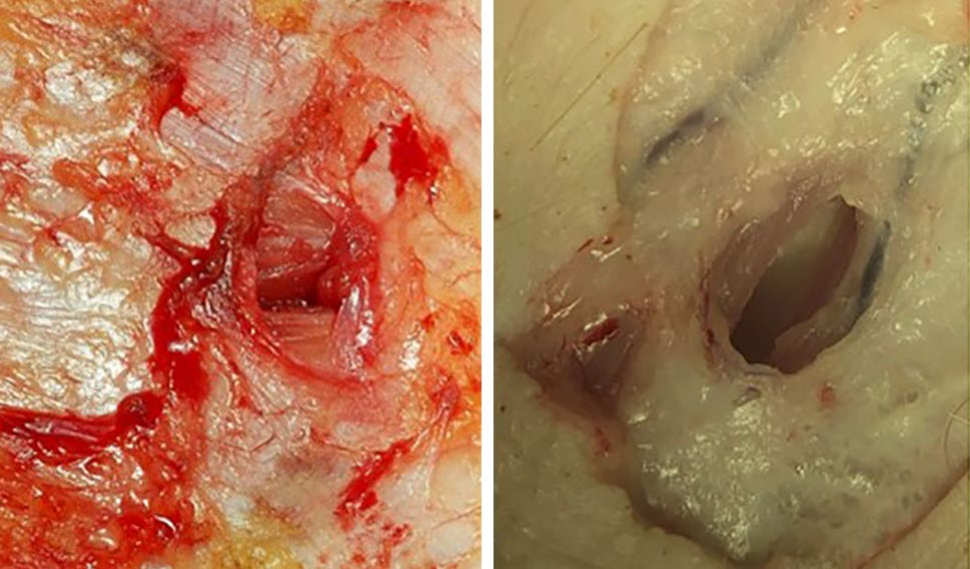
Caused defect size after trocar placement in human (left sided) and in a Porcine Model (right sided)

As study limitation, when the trocars were placed the animals were already euthanized. Thus, no blood perfusion of the tissue took place. This may influence the defect size due to less wound oedema. The trocars were inserted for 60 min. During this time the trocars were not moved. One could argue that a permanent motion of the trocars during surgery may further widen the defect size. Further study protocols should include frequently permanent trocar movement. From experience with tissue and organ removal, it is known that the tissue and muscle structure changes immediately after the death of the animal. Dehydration can lead to tissue shrinkage. It is conceivable that this influenced our defect measurement. Finally, a Porcine Model only allows limited conclusions to be drawn out in human beings. However, fascial defects after trocar placement in humans look similar to those seen in a Porcine Model (Fig. 6).

## Conclusion

Bladed and bladeless trocars do not differ in terms of caused fascial defect size in the Porcine Model at hand. The occurrence of a trocar site hernia might be largely independent from trocar design. Further studies comparing also different trocar tip shapes (conical or pyramidal) are mandatory.
